# Polypropylene/Ethylene—And Polar—Monomer-Based Copolymers/Montmorillonite Nanocomposites: Morphology, Mechanical Properties, and Oxygen Permeability

**DOI:** 10.3390/polym13050705

**Published:** 2021-02-26

**Authors:** Juan Felipe Castro-Landinez, Felipe Salcedo-Galan, Jorge Alberto Medina-Perilla

**Affiliations:** Grupo de Materiales y Manufactura CIPP—CIPEM: Mechanical Engineering Department and Chemical Engineering Department, Universidad de los Andes, Bogota 111711, Colombia; jf.castro1218@uniandes.edu.co (J.F.C.-L.); fesalced@uniandes.edu.co (F.S.-G.)

**Keywords:** polypropylene, ethylene polar monomer-based copolymers (EVA and EVOH), montmorillonite, nanocomposites, oxygen permeability

## Abstract

This research reports the influence of polar monomer contents in ethylene vinyl acetate copolymer (EVA) and ethylene vinyl alcohol copolymer (EVOH) on the morphology, mechanical and barrier properties of polypropylene/ethylene copolymer (PP) reinforced with organically modified montmorillonite (MMT). PP/EVA and PP/EVOH (75/25 wt %) blends were reinforced with 3 wt % MMT in an internal mixer system. Samples were compression-molded into films of 300 μm. The structural characterization was made using X-ray diffraction (XRD), scanning electron microscopy (SEM) and transmission electron microscopy (TEM), the mechanical properties were obtained by tension tests and the barrier properties by oxygen transmission rate (OTR). XRD patterns showed a combination of intercalated/exfoliated morphologies for the MMT, with higher d-001 interplanar distance increments for the blends with higher content of polar functional groups. SEM and TEM micrographs complement the results of the XRD analysis and show differences in the morphologies depending on the miscibility of the polyolefin and the polar monomer copolymer. Mechanical properties and oxygen permeability of composites exhibited a higher improvement, by the addition of MMT, for higher intermolecular interactions and most miscible polymeric system of the EVA. These results show that the higher the number of interactions, given by the VA or OH polar functional groups, the morphology and the miscibility between polyolefin and copolymer imply dispersion improvements of the nanocomposites and, in consequence, a higher improvement on the mechanical and barrier properties of the composite material.

## 1. Introduction

Controlling oxygen permeability through different materials is paramount for the food packaging industry. Due to their combination of higher technical performance and low production costs, polymers are attractive materials to this industry and are the largest used materials for packaging applications [1]. Polymer films having high enough barrier properties are vital for food packaging applications to protect foodstuffs from oxidation and contamination [2,3]. Polymeric materials have a wide range of oxygen permeability, mainly depending on their structural characteristics, such as crystallinity, and the polymer-permeant intermolecular interactions [4]. Polyolefins are the most used polymers on packaging applications, in spite of having a high permeability to oxygen. With the intention of improving the oxygen permeability of polyolefins, some solutions involving different processing techniques, such as multi-layer co-extrusion or coatings, have been developed. However, these processes are complex and expensive and, most importantly, their final products are difficult to recycle [2,3]. For these reasons, mono-layer alternatives based on the incorporation of different non-polymeric substances to the polymer matrix have been extensively investigated in recent times. The two most common of them are involved in the incorporation of oxygen scavengers (active packaging) and the addition of nano-filler structures (passive packaging), in which the dispersion of the filler is the key factor to enhance barrier properties [2,3,5].

Polyolefins reinforced with inorganic layered silicate fillers, such as organically modified montmorillonite (MMT) clay, have demonstrated to improve oxygen barrier [6,7,8,9,10]. To obtain these results, intercalated/exfoliated MMT structures are needed, since they generate tortuous pathways that interfere with the gas molecules diffusion through the polymeric film [6]. Accordingly, the challenge for these kinds of systems is to achieve good clay dispersion [9,10,11]. Several authors have explored the effect of processing conditions (varying the equipment, rotational speed, and residence time) to obtain exfoliated structures [2,4,5,6,11]. However, favorable intermolecular interactions between clay layers and polymer chains have proven to be a key factor to obtain high clay exfoliation; in this sense, strongly non-polar polyolefin chains and their unfavorable interactions with polar clay layers are a challenge to overcome [6,12]. Hence, different functional polyolefin-based polar copolymers are typically added to the polyolefin matrix in order to promote more favorable intermolecular interactions between the polymer chains and the clay layers looking for better nanocomposites morphologies. Using polyolefin-maleic anhydride (MAH) graft, copolymers have shown to produce intercalated structures with fairly good barrier properties [8,9,10,13].

Another less explored approach to improve polyolefin oxygen barrier properties is to blend them with polymers having lower oxygen permeability. Polymer blends are more typically used to obtain improvements in mechanical, thermal, and electrical properties. Polyolefin systems have been blended with several ethylene- and polar-monomer-based copolymers [14], such as ethylene vinyl acetate (EVA) and ethylene vinyl alcohol (EVOH). EVA has typically been blended with different grades of polypropylene (PP) mainly to try to overcome its well-known toughness issues. Blending EVA with isotactic PP (i-PP) has shown to increase PP elongation at break from 25% to 125% although reducing its stiffness [15]. By using a polypropylene/ethylene copolymer—rather than an iPP homopolymer—for the blends with EVA, an equivalent EVA addition further improved the elongation at break (from 65% to 250%) [16,17].

In turn, PP has been blended with EVOH trying to improve the oxygen barrier of the polyolefin. EVOH is well known as a high oxygen permeability polymeric material; between 0.4–3 cm^3^ mm/m^2^ day atm [18]. The good oxygen barrier obtained for this polymer blend is mainly due to its highly crystalline structures and its strong inter- and intra- molecular bondings caused by the polar hydroxyl groups present in the vinyl alcohol unit [18]. The use of proper compatibilizers in these blends has been shown to be very relevant. EVOH addition improved iPP permeability by 24% without using a compatibilizer, and when better PP-EVOH interactions are promoted by using sodium ionomer, maleate ester, or maleic anhydride, improvements up to 60–90% were seen [19,20]. The inclusion of these compatibilizers promoted the interfacial interactions between polymers, changed the morphological structures of the blend enhancing both barrier and mechanical properties: with 40% EVOH addition, the Young modulus increased from 854 MPa to 1150 MPa [21,22,23,24].

Combining the two approaches discussed above to try to improve polypropylene oxygen barrier properties, i.e., (i) the incorporation of exfoliated layer silicates and (ii) blending it with a polymer with better barrier properties, is the key contribution of this work. Hence, PP/EVA/MMT and PP/EVOH/MMT ternary composites were evaluated. From the nanocomposites point of view, these two ethylene- and polar-monomer-based copolymers (EVA and EVOH) have a much higher number of polar functional groups than the typically used polyolefin-MAH-grafted copolymers, making them good candidates to produce exfoliated MMT structures. In the case of the binary EVA/MMT nanocomposites, mainly exfoliated structures with intercalated tactoids have been reported [25,26,27,28], whereas for the binary EVOH/MMT nancomposites, intercalated structures with small exfoliation are seen [29,30,31,32]. In this way, according to MMT exfoliation, better barrier properties are expected for the PP/EVA/MMT nanocomposites. From the polymers blend perspective, PP/EVOH/MMT systems are expected to performed better than the PP/EVA/MMT, due to EVA have much higher oxygen permeability (in the order of 800–1000 cm^3^ mm/m^2^ day atm).

PP/EVA/MMT nanocomposites have been extensively studied mainly to evaluate their mechanical properties, reporting an improvement in stiffness but compromising toughness of the blends [33,34,35,36]. However, oxygen permeability properties for these systems have, to the best of our knowledge, not been investigated. The ethylene- and polar-monomer-based copolymers selected here allow to compare the effects of the different nature of each polar functional group on the morphology, mechanical, and oxygen permeability properties of the composites. The variation of polar monomer content for both cases was also evaluated, expecting more exfoliated clay structures for the composites having a higher number of polar functional groups [6,12]. The aim of this work is to investigate oxygen permeability and validate mechanical properties (stiffness and elongation at break) in PP/EVA/MMT and PP/EVOH/MMT nanocomposites and its dependency on the (polymers blend and clays) morphology obtained based on the number and types of polar functional groups.

## 2. Materials and Methods

A propylene-ethylene random copolymer (PP) (Esenttia 01R25) with a melt flow index of 0.8 g/10 min and density of 0.9g/cm^3^ was used. Ethylene vinyl acetate (Elvax660) with 12 wt % of vinyl acetate monomer content (EVA12), with a melt flow index of 2.5 g/10 min and density of 0.93 g/cm^3^, was purchased from DuPont; EVA28, with 28 wt % of vinyl acetate monomer content (Evatane2805) and a melt flow index of 5 g/10 min and density of 0.95 g/cm^3^, was purchased from Arkema. Ethylene vinyl alcohol (EVOH38), with 38 mol % of ethylene monomer content and a melt flow index of 1.7 g/10 min and density of 1.17 g/cm^3^, and EVOH44, with 44 mol % of ethylene monomer content and a melt flow index of 5.7 g/10 min and density of 1.14 g/cm^3^ (Eval H171 and E105, respectively) were both kindly supplied by Kuraray. Polypropylene grafted maleic anhydride (PP-g-MAH) (Orevac18760), with a melt flow index of 3.8 g/10 min and density of 0.91 g/ cm^3^, was purchased from Cromptom. Finally, organo-modified montmorillonite (MMT) (Nanofill SE3000), modified with hydrogenated di-tallow di-methyl quaternary ammonium salt, specific weight of 1.2 cm^3^/g and average particle size D_50_ of 8 µm, was supplied by Süd Chemie AG.

Prior to melt-blending, all the materials were dried: PP, EVA12, EVA28, PP-g-MAH and MMT for 24 h at 100 °C; EVOH38 and EVOH44 for 6 h at 90 °C. A masterbatch (MA) to carry the MMT was manufactured mixing the PP-g-MAH and MMT (80/20 wt %) in a twin screw extruder Brabender Plasti-Corder PLE331 with rotational speed of 100 rpm and a temperature profile of 190, 195, 200, 205 and 220 °C.

For the evaluation of the type of copolymer on the nanocomposites, blends of PP with EVA12, EVA28, EVOH38 and EVOH44 (75/25 wt % polyolefin/copolymer) and a constant addition of 3 wt % of MMT [7,8,37] were prepared using an internal mixer at 190 °C rotational speed of 120 rpm and residence time of 300 s after torque stabilization were achieved. Neat polymers and polymer blends were processed at the same condition as control samples. In Table 1, the samples used are shown and their designation are introduced. The polymer blends were milled, with a high-speed blade mill, at −80 °C to a particles of size of 2 mm, after which they were compression molded to films with 300 µm thickness (temperature of 190 °C, pre-heated for 12 min, pressed at 1.5 MPa for 1 min and at 11 MPa for 1.5 min and cooled with water at room temperature for 10 min).

### 2.1. Dispersion and Morphologies of the Composites

For evaluating morphologies achieved for the nanoparticles (MMT) within the polymeric matrix a transmission electron microscope (TEM), JEOL JEM 1400Plus, operated at 80 kV, was used. The nanocomposites samples of Table 1 containing clay were ultramicrotomed, with an ultramicrotome Leica EM UC7, to 70 nm thickness.

The morphology of the polymeric blends was characterized via scanning electron microscopy (SEM). Each sample, containing PP and polar copolymer (EVA or EVOH), was cryo-fractured in liquid nitrogen at −160 °C, then sputter coated with gold prior to examination under JEOL JSM 6490LV, operated at 12 kV.

X ray diffraction (XRD) patterns were recorded with an Ultima III Rigaku equipment for all the samples in Table 1, with a wavelength of λ= 0.154 nm. Wide angle X-ray scattering (WAXS) analysis was performed at a rate of 0.05°/min from 2° to 30° 2θ. The interplanar distance of the plane 001 plane (*d*) of the MMT in each sample was calculated with Bragg’s law of diffraction (Equation (1)) and using the principal peak of the XRD patterns:

(1) nλ=2dsin(θ)

### 2.2. Degree of Cristallinity of Polymers

The degree of crystallinity of each specimen was analyzed by differential scanning calorimetry (DSC) using a TA Instruments Q200 DSC. About 8 mg of each sample was scanned in a cycle of heating-cooling-heating from −50 to 200 °C at a rate of 5 °C/min, for both heating and cooling rates. The second heating scan was used to determine the degree of crystallinity ($\chi_{c}$) according to Equation (2), where ΔH is the melting enthalpy of the sample, $\Delta H_{m}^{0}$ is the melting enthalpy of the 100% crystalline polymer matrix and *y* is the total weight percentage of nanoclay and blend component for a two polymers blend [11]. The melting enthalpy was considered as 201.1 J/g for PP [11], 158.6 J/g for EVOH [37] and 293.0 J/g for EVA, for the ethylene polar copolymers the enthalpy varies with the co-polymer monomer content [38].

(2) $$\chi_{c}=\frac{\Delta H}{\Delta H_{m}^{0}\cdot \left(1-y\right)}\times 100$$

### 2.3. Barrier Properties of the Nanocomposites and Polymer Blend

Oxygen permeability of compression molded-films was measured using an oxygen transmission rate (OTR) tester, Mocon OX-TRAN 2/21. The ASTM D3985 Standard, Test Method for Oxygen Gas Transmission Rate Through Plastic Film and Sheeting Using a Coulometric Sensor, was used for the determination of the permeability of the samples. The test was performed at 23 °C and a pressure of 558 mmHg. Aluminum foil was used as an area reducer (5.06 cm^2^) for the samples tested. The reported value of permeability is the average of three different permeability measurements.

### 2.4. Mechanical Properties of the Nanocomposites and Polymer Blends

Mechanical properties were evaluated by a tensile test; rectangular-shaped specimens (25.4 mm × 101.6 mm × 0.3 mm), as per ASTM D882 Standard, Test Method for Tensile Properties of Thin Plastic Sheeting, were evaluated in an Instron 3663 universal testing machine with a cross head speed of 50 mm/min and a gauge length of 50 mm in all the tests. The results of the Young modulus, elongation at break and tensile strength were reported as the average of six specimens of each sample.

## 3. Results and Discussion

### 3.1. Polymeric Blends and Nanocomposites Morphologies and Structures

The morphology of blends and nanocomposites systems has a close relationship with the permeation and the mechanical properties of the materials. The crystallinity degree of the neat materials, blends and nanocomposites, obtained by DSC (representative thermograms for each sample are shown in Figures S1–S4 in supplementary materials) and Equation (2), is shown in Table 2 for each polymer. With the DSC technique, it is possible to differentiate the crystalline domains of PP and polyethylene (EVA and EVOH) because of their different melting and crystallization temperatures [11,39,40,41]. Thus, the appearance of both peaks for the polymers blended indicate that there are two phases that are immiscible but compatible. The results indicate that the addition of EVA on a PP matrix decreased the initial crystallinity of the PP phase from 34% to approximately 16% due to the introduction of a high amorphous polymer lowering the chemical potential of crystallization of both polymers [42], while for the PP/EVOH blends, the crystallinity is not significantly affected. DSC showed that the crystalline phases of the polymers are distinct and each polymer has an independent crystallization process [40,43]. The crystallinity degree of a polymer is an important factor on the study on permeability, since the mass transport phenomena takes place in the amorphous phases of the polymers [4,44,45]. Moreover, the mechanical properties of the polymers could be defined by its final morphology, in this case, a reduction of the crystalline part of the matrix would lead to an improvement of the ductility [35].

Table 2 also shows the degree of crystallinity of each polymer phase for the different systems after the addition of 3 wt % of MMT. Comparing the PP/EVA and PP/EVOH blends to its corresponding nanocomposite, there is no increment of the crystallinity for the PP phase nor for the PE phase. The last result is expected on these types of blend materials and this amount of clay aggregated; it is important to note that the addition of MMT in similar systems does not have a significant effect on the degree of crystallinity of the polymers [44,45].

The melting temperature is also reported in Table 2, showing a slight increase of such a property for the individual polymers. Contrary to what has been reported for PP/EVA and PP/EVOH blends, the melting point of the polymers did not decrease [16,17,42], except for the PP/EVOH38/MMT nanocomposite. The variation of the melting points for both systems (PP/EVA and PP/EVOH) evaluated in the polymer blends and nanocomposite is between 3–7 °C, which indicates that there is no phase separation [16].

**Figure 1:**
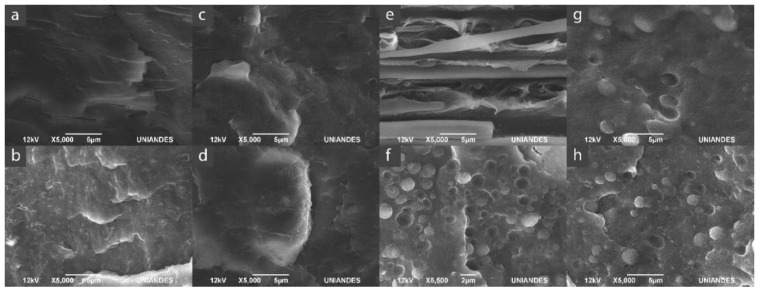
SEM images for the polymeric blends and the nanocomposites. (**a**) PP/EVA12, (**b**) PP/EVA12/MMT, (**c**) PP/EVA28, (**d**) PP/EVA28/MMT, (**e**) PP/EVOH38, (**f**) PP/EVOH38/MMT, (**g**) PP/EVOH44 and (**h**) PP/EVOH44/MMT.

The SEM micrographs of the neat polymer blends and the nanocomposites are shown in Figure 1. The phases of polymer materials were identified by their electron density, which is greater for the copolymers than the polyolefin, making them brighter on the SEM micrographs. It is well known in the literature that both blends are immiscible but compatible, forming a spherical dispersed phase in the polyolefin matrix (using the concentrations of PP and ethylene- and polar-monomer-based copolymer proposed in this research). This always occurred unless a compatibilizer is added where a co-continuous phase can be formed [35,42,46,47]. Comparing Figure 1a,c, the micrographs clearly show three different morphologies: a co-continuous phase for the PP/EVA systems (regardless the content of VA), a spherical- shaped dispersed phase for PP/EVOH44 (Figure 1g) and a fibrillar shape dispersed phase for PP/EVOH38 (Figure 1e). This shows that the compatibility of PP/EVA systems is higher than the PP/EVOH ones. The morphology differences between the PP/EVA and the PP/EVOH systems can be attributed to two factors. The first one is the viscosity ratio between the polymers $\eta_{c}=\eta_{PP}/\eta_{EVA/EVOH}\;$. In the case of PP/EVA, this ratio should be closer to the co-continuous phase limit; thus, the morphologies achieved are not segregated, while the PP/EVOH systems should have a ratio higher than the limit. Hence, dispersed phases of EVOH appear [19,22,46,48]. The second factor is the compatibility of PP with each copolymer, which is greater for PP/EVA than PP/EVOH, since these systems have larger amounts of ethylene comonomer content: 88% for EVA12 and 72% for EVA28 against 38% for EVOH38 and 44% for EVOH44. These higher amounts of ethylene comonomer will generate more favorable interactions with the propylene and ethylene segments of the PP matrix [16,17,49,50]. Polymer blends morphology have a significant role in properties as homogenously distributed barrier layers or droplets can affect the permeability mechanisms of the blends [3]. On the other hand, the difference in the EVOH morphologies is more related to the manufacturing process. As the EVOH38 copolymer has a smaller content of ethylene monomer in its composition, particles tend to deform easily by the film manufacturing process [20,48,51].

The SEM micrographs in Figure 1b,d,f,h show the effect of the addition of MMT on the morphology of PP/EVA and PP/EVOH blends. In the PP/EVA12/MMT, PP/EVA28/MM, and PP/EVOH44/MMT composites (Figure 1b,d,h), the morphology of the polymer phases do not show any change (size or shape) compared with the respective blends (Figure 1a,c,g). On the other hand, for the EVOH38 nanocomposite (Figure 1f), it is evident that the shape of the dispersed phase changes from fibrillar to spherical. This means that the addition of the MMT to the PP/EVOH system with high concentrations of polar co-monomer decreases the interfacial tension between the dispersed phase of EVOH38 and the PP [20,34,35].

**Figure 2:**
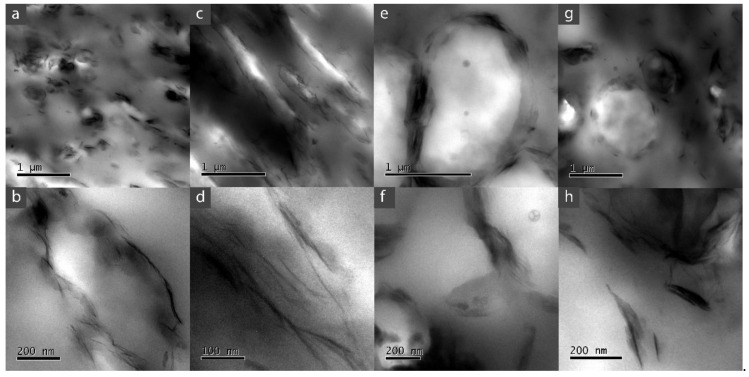
TEM images for the polymeric blends and the nanocomposites. (**a**,**b**) PP/EVA12/MMT, (**c**,**d**) PP/EVA28/MMT, (**e**,**f**) PP/EVOH38/MMT and (**g**,**h**) PP/EVOH44/MMT.

TEM images of the nanocomposites are shown in Figure 2. MMT in the nanocomposites shows a combination of morphologies between intercalated and exfoliated structures. As expected, for PP/EVA/MMT nanocomposites the MMT have greater dispersions, exhibiting clay tactoids (Figure 2a,c) and even some individual platelets (Figure 2b,d), than in PP /EVOH/MMT systems, where just tactoid structures of MMT appear (Figure 2f,h) [52]. The structures and dispersion achieved agreed with the obtained by other investigations. In addition, different clay structures in PP/EVA systems, i.e., intercalated and exfoliated, tend to be dispersed in the EVA phase [33,35]. Particularly, a greater exfoliation and dispersion of the filler (MMT) was found in the EVA28 system rather than the EVA12 system (compare Figure 2b,d). This result was also expected, as the former has a greater content of vinyl acetate polar groups—promoting the interaction with the clay and consequently its exfoliation—than the latter. On the other hand, in PP/EVOH systems, agglomerate structures are mostly seen in the PP phase and a few intercalated/exfoliated structures were found in the interphase of copolymer and polyolefin; the MMT seems to be surrounding the EVOH phases in these blends, especially in the EVOH38 sample (Figure 2e,g). This explains the dispersed phase shape change seen, from fibrillar to spherical, when comparing these composites to the respective neat polymer blend (Figure 1f,e); in PP/EVOH/MMT composites, the clays, being polar, prefers to stay away from the PP matrix, looks for the PP-EVOH interface and changes its shape.

**Figure 3:**
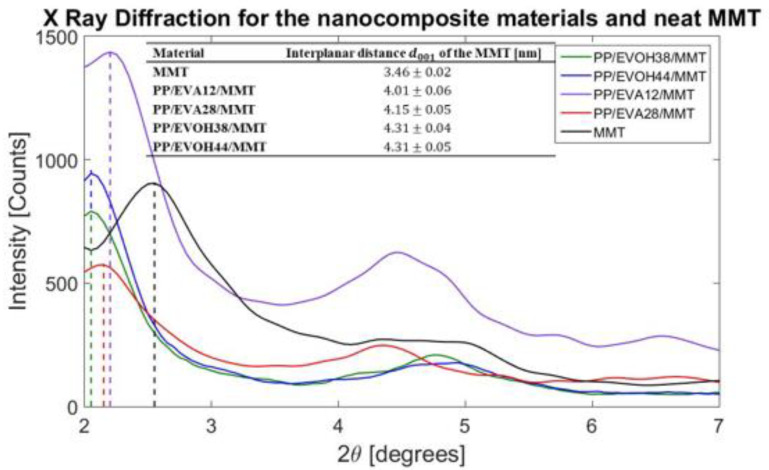
XRD for the MMT and the nanocomposite materials manufactured with different copolymers for the determination of d-space of plane 001 of the clay.

The XRD results of the nanocomposites are shown in Figure 3. Comparing the interplanar distance of the 001 plane of the MMT in the nanocomposites with the neat MMT, an intercalation of the clay is evident in all the composites, caused by the introduction of some polymer chains into the clay galleries. PP/EVOH/MMT composites achieved a somewhat higher interplanar distance of the tactoids than PP/EVA/MMT composites. These results, in addition to the TEM images, show that PP/EVOH blends have a significant interaction with MMT galleries, but not strong enough in order to break the Van der Waals bonds between the layers and produce exfoliated clay structures. Particularly, the largest clay gallery distance (4.31 nm) is achieved by the composites made of EVOH copolymer, regardless of the ethylene comonomer content. In the composites made of EVA copolymer, the interplanar distance is affected by the content of vinyl acetate comonomer; as expected, the increase of polar comonomer content achieved larger clay interlayer distances [8,33,36,50]. The interplanar distance is an important factor that can affect the tortuosity pathway for the oxygen molecules through the nanocomposite material; this distance is an indicator of the intercalation level of the composite [53,54]. It is important to note that the differences of the intensity of the peaks are explained by a possible different orientation of the clay platelets on the composite [55].

### 3.2. Oxygen Permeability and Mechanical Properties

**Figure 4:**
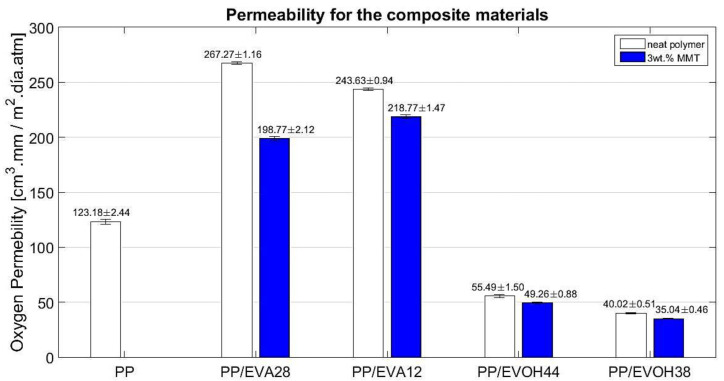
Oxygen permeability for the neat PP, polymeric blends PP/EVA and PP/EVOH and nanocomposite materials made of the different kinds of copolymers.

As shown in Figure 4 PP/EVA blends have higher oxygen permeability than PP, comparatively 116% and 98% higher with 28 and 12 wt % vinyl acetate content, respectively. PP/EVOH blends have lower permeability than PP, a 55% and 67% comparative reduction for 67 and 72 wt % vinyl alcohol content, respectively. The permeability of the blends varies accordingly to the reference values of the neat EVA and the neat EVOH. The increase of the PP permeability with the addition of EVA is explained by two factors. First, the addition of a copolymer with a higher number of amorphous phases that lead to a decrease of the bulk crystallinity degree of the polymer blend. Second, the interaction of the two polymers, which can affect the crystallization processes of the materials, lowering the chemical potential of the crystalline phase for the two-phase system, changing the crystalline domains of the neat PP (reduction of 50% as seen on the DSC results, (Section 3.1.) due to the addition of EVA). On the other hand, the decrease in the permeability of the PP due to the addition of EVOH is explained by the addition of a material with a low oxygen permeability due to the intermolecular interactions and a higher degree of crystallinity, generating separated phases of polymers and a different pathway for the oxygen molecules in each polymeric phase [50].

The results of permeability of the polymeric blends with the addition of 3 wt % of MMT are also shown in Figure 4. The permeability of the nanocomposite’s materials decreased compared with the polymeric blends in every single case. The last results indicate that the addition of MMT generated an increase of the tortuosity factor, leading to a decrease in the permeation. However, there seems to be a greater decrease in permeability (of 26%) for the EVA28 system compared to the decreases for the EVA12, EVOH38 and EVOH44 systems (10%, 11% and 12%, respectively). It has been reported that the presence of MMT in the composite causes a decrease in permeability properties of the polymers due to the more tortuous paths for the diffusing molecules [2,53,54]. Particularly, exfoliation of the systems has been reported as an important factor to determine the permeability of a nanocomposite [45,56]. This evidence shows that there are two important factors that affect the permeation of these kinds of ternary systems (polyolefin/copolymer/clay). The first factor is the crystallinity of the polymeric blend systems; the second factor is the tortuosity pathway generated by the addition of MMT. In EVA systems (the most miscible with PP as verified on SEM and TEM), the higher content of monomer of polar functional group increased the intermolecular interactions between polymers and clay, leading to more exfoliated systems and a greater improvement of permeability properties. On EVOH systems, the last interactions do not have a significant effect on the oxygen permeability, even though in the EVOH composite materials, the number of polar functional groups is higher compared than the EVA composite materials. Instead, the MMT tends to interact mostly with the higher polar copolymer (dispersed phase) leading to the weakest interaction with the PP matrix and a lower improving of permeability to oxygen in the PP/EVOH composite materials. Additionally, in EVOH systems, the dispersed phase can lead to free paths on the interphases, which can be used by the oxygen molecules to diffuse easily on the polymeric film [45].

Mechanical properties of the PP are also affected by the addition of the copolymers. The Young modulus, tensile strength and the elongation at break are shown in Table 3 (a representative stress-strain curves for each sample configuration is shown in Figures S5 and S6). The PP/EVA blends Young modulus is 0.3 GPa lower compared to the pure PP material. For the PP/EVOH blends, the Young modulus is 0.1 GPa higher than for pure PP. Young modulus is not affected in any blended system by the polar monomer copolymer content. The change of the stiffness of the materials is due to the nature of each polymer added to the systems, and the blends follow a rule of mixtures for stiffness [39,57]. The elongation at break for the blends varies considerably compared to the neat PP; by adding the EVA12, there is an increment of 135%, while by adding EVA28, the increment is 210%, approximately. In the case of EVOH38 and EVOH44, the reduction is approximately 680% and 670%, respectively. These changes are explained by the blending dispersion and morphologies obtained. For PP/EVA a co-continuous phase created with a more ductile material will lead to an increment of the elongation at break, while a dispersed phase, such as what was obtained in the PP/EVOH systems, tend to have an early breaking point.

The stiffness, tensile strength and elongation at break of the nanocomposites are shown in Table 3. With the addition of 3 wt % of MMT, the stiffness of the blends materials has a greater improvement on the EVA systems (21% and 15% for blends with EVA28 and EVA12, respectively, compared with the corresponding polymeric blend) than EVOH systems. For the EVOH44 and EVOH38 blends, the improvement of the stiffness was only by 0.8% and 10.2% respectively; a fact that can be explained by the morphologies obtained in the blends and the addition of a stiffer copolymer (EVOH) than EVA [39,45]. These results show that the MMT has a better interaction with the PP/EVA blends, as they act like a greater reinforcement than the effect on the PP/EVOH blends. Interestingly, the elongation at break and the tensile strength showed no significant change with the incorporation of the clay into the polymeric blends for both PP/EVA and PP/EVOH systems. Contrary to what is typically reported in the literature, stiffness of the polymeric blends improved and the elongation at break remained the same [35,37]. In these kinds of systems, the modification of the stiffness and the elongation at break is mostly related with the polymeric blends (morphologies and polymer nature), rather than the effect of the MMT in the composite with the addition of the studied concentration of clay [8,16,17,42].

## 4. Conclusions

Both polymer blend systems (PP/EVA and PP/EVOH) are compatible but not miscible, showing a co-continuous phase for PP/EVA and a disperse separated phase for PP/EVOH. The morphologies obtained are different due to the different intermolecular interactions of the PP with the respective ethylene- and polar-monomer-based copolymer. By adding MMT to PP/EVOH systems, the morphology of the blends is changed from a fibrillar to spherical shape, but the clay is agglomerated in the interphase between the dispersed phases and the PP. For the PP/EVA, the morphology of the blends is not affected by the MMT. In PP/EVA, the clay dispersion is better (tendency to higher clay exfoliation) compared to the PP/EVOH system. The increment of the number of favorable intermolecular interactions between MMT and the polymeric chains (with higher contents of functional polar groups, such as in the case of EVA composites) will produce a more exfoliated structure.

For the mechanical properties, the addition of MMT in the polymer blends produced an improvement of the Young modulus. Moreover, the elongation at break and tensile strength of the blends have no significant change by adding the MMT, so the toughness of the polymer blend is also not significantly affected. This demonstrates that the addition of MMT at the levels studied in this research acts as a stiffness reinforcement for the polymeric blends regardless of the type of polar functional group of the copolymer.

Barrier properties of the blends are affected by the nature of the functional polymers added. For PP/EVA systems there is an increase of the permeability of the PP compared to the significant reduction of the PP/EVOH system, even having dispersed phases of EVOH. By adding the MMT, PP/EVA systems showed a greater barrier improvement compared to PP/EVOH systems, as a consequence of the fact that for PP/EVA/MMT clays are dispersed through the entire matrix, while for EVOH systems, the MMT is agglomerated in the dispersed phases. Specifically, for PP/EVA, when using a higher number of functional polar groups (EVA28) in the nanocomposite, a greater improvement in permeability is obtained compared to the EVA12. Then, the permeability improvement is explained by the generation of tortuous pathways that modified the oxygen molecules diffusion process through the polymeric material.

## Figures and Tables

**Table 1:** Composition in wt % of the samples prepared.

Materials	Sample Name	Composition [wt %]			
		PP	EVA	EVOH	MMT
**Neat polymers**	PP	100	−	−	−
	EVA12	−	100	−	−
	EVA28	−	100	−	−
	EVOH38	−	−	100	−
	EVOH44	−	−	100	−
**Neat Polymer blends**	PP/EVA12	75.0	25.0	−	0
	PP/EVA28	75.0	25.0	−	0
	PP/EVOH38	75.0	−	25.0	0
	PP/EVOH44	75.0	−	25.0	0
**Nanocomposites**	PP/EVA12/MMT	72.8	24.2	−	3.0
	PP/EVA28/MMT	72.8	24.2	−	3.0
	PP/EVOH38/MMT	72.8	−	24.2	3.0
	PP/EVOH44/MMT	72.8	−	24.2	3.0

**Table 2:** Thermal transitions and degree of crystallinity for the neat polymers, polymeric materials into the blends and nanocomposites. Determined by DSC analysis.

Type of Material	Material	Tm [°C]		Tc [°C]		Degree of Crystallinity [%]	
		PP	Ethylene and Polar Monomer Copolymer	PP	Ethylene and Polar Monomer Copolymer	PP	Ethylene- and Polar- Monomer-Copolymer
**Neat Polymers**	PP	142.91	−	102.98	−	34 ± 2	−
	EVA12	−	86.82	−	72.05	−	14 ± 1
	EVA28	−	73.38	−	54.22	−	22 ± 1
	EVOH38	−	162.55	−	142.71	−	37 ± 2
	EVOH44	−	157.11	−	137.75	−	34 ± 2
**Neat Polymer blends**	PP/EVA12	143.08	87.37	105.84	71.47	17 ± 1	10 ± 1
	PP/EVA28	143.91	78.40	102.13	54.23	15 ± 1	3 ± 1
	PP/EVOH38	147.97	164.55	114.85	143.40	38 ± 2	31 ± 1
	PP/EVOH44	148.45	164.65	114.64	138.60	38 ± 1	28 ± 1
**Nanocomposites**	PP/EVA12/MMT	144.71	87.19	107.24	71.57	15 ± 1	10 ± 1
	PP/EVA28/MMT	145.34	77.88	105.42	56.24	16 ± 1	3 ± 1
	PP/EVOH38/MMT	148.54	159.18	115.47	142.70	28 ± 2	28 ± 1
	PP/EVOH44/MMT	148.01	159.56	115.30	140.40	34 ± 1	31 ± 1

**Table 3:** Mechanical properties (Young modulus, elongation at break and tensile strength) of polymeric blends and nanocomposites samples.

Type of Material	Material	Young Modulus [GPa]	Elongation at Break [%]	Tensile Strength [MPa]
**Neat Polymers**	PP	0.96 ± 0.04	690 ± 10	22.30 ± 4.00
	EVA12	0.10 ± 0.05	825 ± 60	14.88 ± 3.27
	EVA28	0.09 ± 0.04	900 ± 50	16.24 ± 4.21
	EVOH38	2.32 ± 0.21	43 ± 21	57.41 ± 3.12
	EVOH44	1.83 ± 0.23	48 ± 18	45.05 ± 7.34
**Polymeric blends**	PP/EVA12	0.65 ± 0.04	587 ± 45	17.62 ± 0.64
	PP/EVA28	0.60 ± 0.01	310 ± 42	16.85 ± 0.63
	PP/EVOH38	1.16 ± 0.05	6 ± 2	20.28 ± 2.51
	PP/EVOH44	1.13 ± 0.04	16 ± 7	19.01 ± 1.89
**Nanocomposites materials**	PP/EVA12/MMT	0.76 ± 0.02	574 ± 58	17.89 ± 0.61
	PP/EVA28/MMT	0.75 ± 0.01	353 ± 55	17.42 ± 0.45
	PP/EVOH38/MMT	1.29 ± 0.02	13 ± 8	19.95 ± 1.05
	PP/EVOH44/MMT	1.15 ± 0.04	19 ± 10	17.94± 0.83

## Data Availability

The data presented in this study are available in supplementary materials mentioned before.

## Supplementary Materials

The following are available online at https://www.mdpi.com/2073-4360/13/5/705/s1, Figure S1: DSC thermogram in the heating stage for the neat polymeric materials (PP, EVA28, EVA12, EVOH44 and EVOH38) used for the compositions. The melting temperature (Tm) and the melting enthalpy (Hm) are displayed. The different curves have an offset +Z from zero to have a better comparison. Figure S2: DSC thermogram in the cooling stage for the neat polymeric materials (PP, EVA28, EVA12, EVOH44 and EVOH38) used for the compositions. The crystallization temperature (Tc) and the crystallization enthalpy (Hc) are displayed. The different curves have an offset +Z from zero to give a better comparison. Figure S3: DSC thermogram in the heating stage for the neat polymeric blend (solid lines) and nanocomposite (dotted lines) materials manufactured. The crystallization temperature (Tm) and the crystallization enthalpy (Hm) are displayed. The different curves have an offset +Z from zero to give a better comparison. Figure S4: DSC thermogram in the cooling stage for the neat polymeric blend (solid lines) and nanocomposite (dotted lines) materials evaluated. The crystallization temperature (Tc) and the crystallization enthalpy (Hc) are displayed. The different curves have an offset +Z from zero to give a better comparison. Figure S5: Stress-Strain curves for the neat polymers PP, EVA28 and EVA12 (Solid lines), polymeric blend PP/EVA28 and PP/EVA12 (pointed lines) and nanocomposites PP/EVA28/MMT and PP/EVA12/MMT (dotted lines). Representative curves out of a batch of 10 samples per composition. Figure S6: Stress-Strain curves for the neat polymers PP, EVOH44 and EVOH38 (Solid lines), polymeric blend PP/EVOH44 and PP/EVOH38 (pointed lines) and nanocomposites PP/EVOH44/MMT and PP/EVOH38/MMT (dotted lines). Representative curves out of a batch of 10 samples per composition.
