# Supplementary material for: Polypropylene/Ethylene—And Polar—Monomer-Based Copolymers/Montmorillonite Nanocomposites: Morphology, Mechanical Properties, and Oxygen Permeability
Source: Polymers (Basel). 2021 Feb 26;13(5):705. doi: 10.3390/polym13050705 (PMC7956292; doi:10.3390/polym13050705)
Supplement: Supplementary file 1 [file polymers-13-00705-s001.pdf]

Supplementary Materials

DSC thermograms

DSC thermograms for the neat materials are shown in the Figures S1 and S2 for heating and cooling stages, respectively. Close to each representative curve there is an indication of the melting temperature and enthalpy used in the analysis of degree of crystallinity of every sample.

Figures S3 and S4 correspond to the DSC of the neat polymer blend materials (solid lines) and the nanocomposites (dotted lines) for heating and cooling stages, respectively.

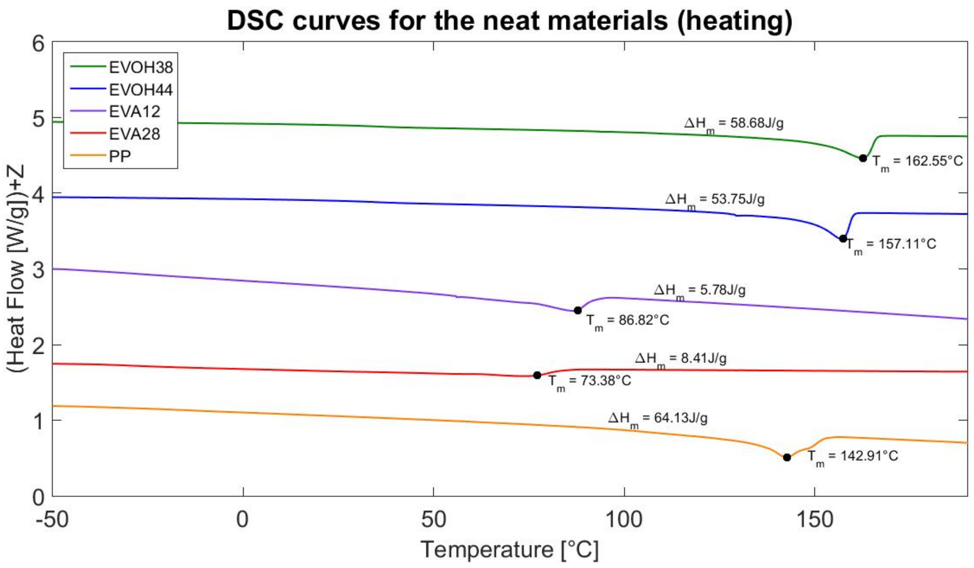

Figure S1. DSC thermogram in the heating stage for the neat polymeric materials (PP, EVA28, EVA12, EVOH44, and EVOH38) used for the compositions. The melting temperature (Tm) and the melting enthalpy (Hm) are displayed. The different curves have an offset +Z from zero to have a better comparison.

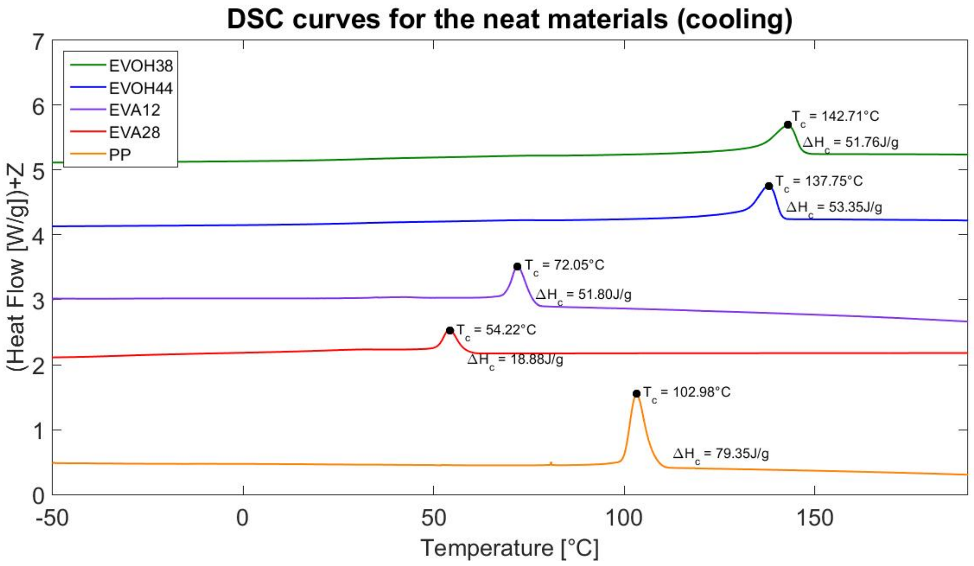

Figure S2. DSC thermogram in the cooling stage for the neat polymeric materials (PP, EVA28, EVA12, EVOH44, and EVOH38) used for the compositions. The crystallization temperature (Tc) and the crystallization enthalpy (Hc) are displayed. The different curves have an offset +Z from zero to have a better comparison.

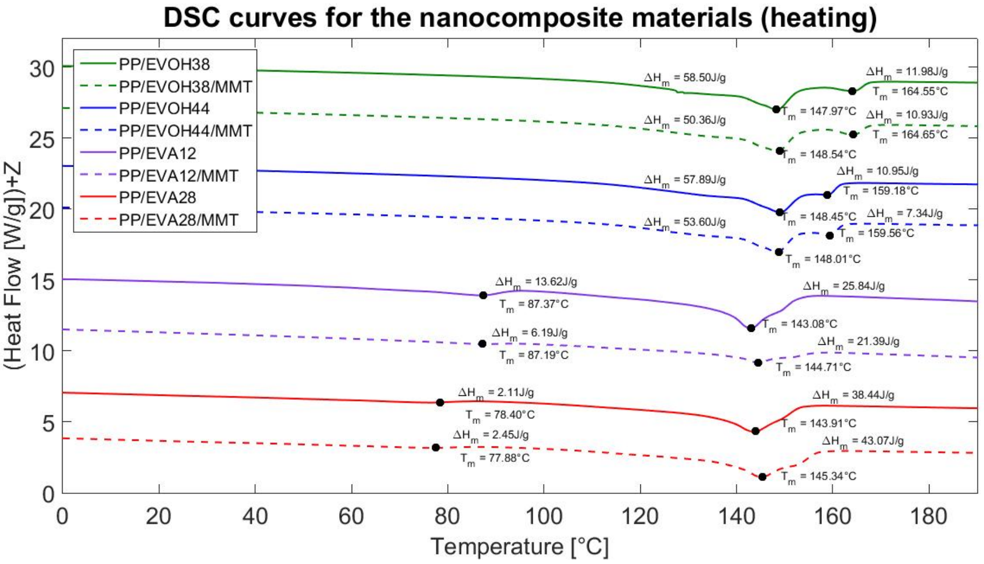

Figure S3. DSC thermogram in the heating stage for the neat polymeric blend (solid lines) and nanocomposite (dotted lines) materials manufactured. The crystallization temperature (Tm) and the crystallization enthalpy (Hm) are displayed. The different curves have an offset +Z from zero to have a better comparison.

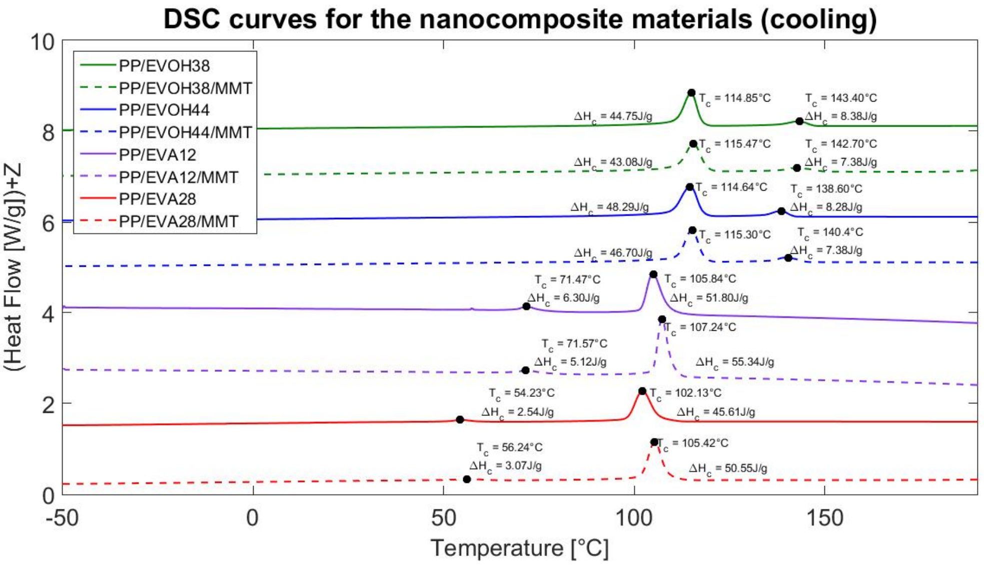

Figure S4. DSC thermogram in the cooling stage for the neat polymeric blend (solid lines) and nanocomposite (dotted lines) materials evaluated. The crystallization temperature (Tc) and the crystallization enthalpy (Hc) are displayed. The different curves have an offset +Z from zero to have a better comparison.

Stress-Strain curves

In the Figures S5 and S6 there are one representative curve of the six samples evaluated per each combination of PP, ethylene – and polar- monomer based copolymers, and montmorillonite. EVA neat materials and its combinations are available in Figure S5 while the EVOH neat materials and its compositions are shown in the Figure S6.

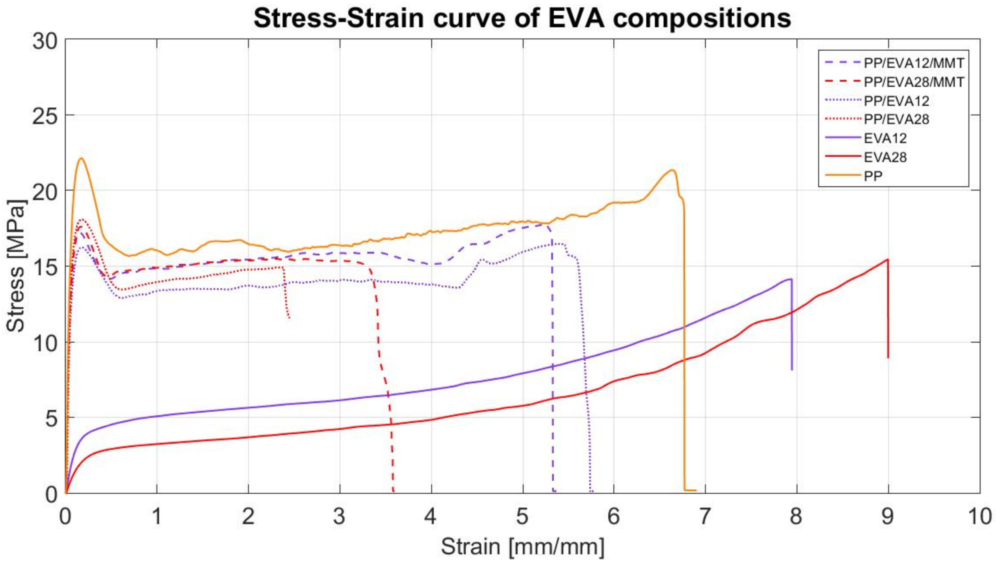

Figure S5. Stress-Strain curves for the neat polymers PP, EVA28, and EVA12 (Solid lines), polymeric blend PP/EVA28 and PP/EVA12 (pointed lines), and nanocomposites PP/EVA28/MMT and PP/EVA12/MMT (dotted lines). Representative curves out of a batch of 6 samples per composition.

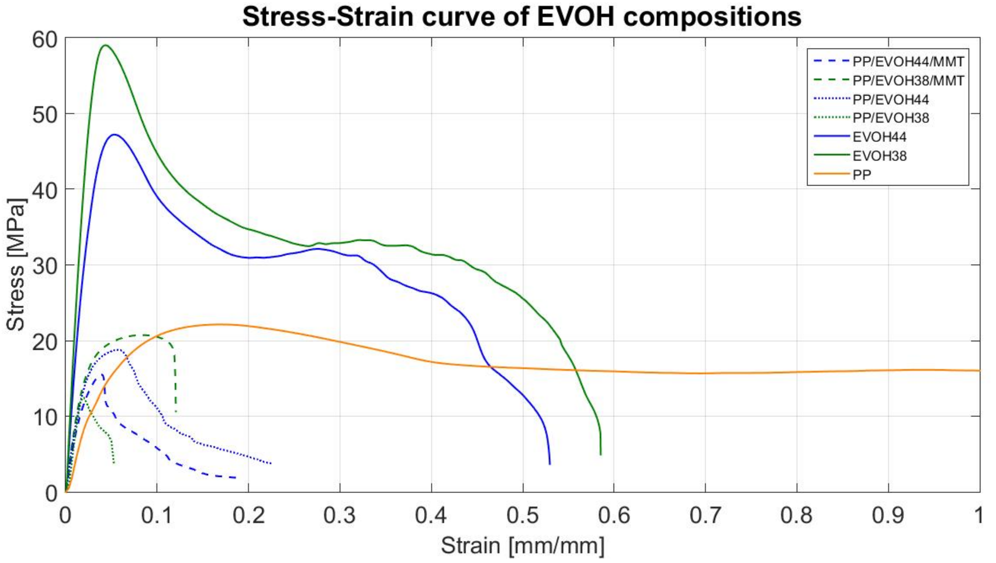

Figure S6. Stress-Strain curves for the neat polymers PP, EVOH44, and EVOH38 (Solid lines), polymeric blend PP/EVOH44 and PP/EVOH38 (pointed lines), and nanocomposites PP/EVOH44/MMT and PP/EVOH38/MMT (dotted lines). Representative curves out of a batch of 6 samples per composition.
